# Reduction of experimental ocular axial elongation by neuregulin-1 antibody

**DOI:** 10.3389/fmed.2023.1277180

**Published:** 2023-10-26

**Authors:** Xu Han Shi, Li Dong, Rui Heng Zhang, Wen Da Zhou, Yi Fan Li, Hao Tian Wu, He Yan Li, Chu Yao Yu, Yi Tong Li, Ya Xing Wang, Jost B. Jonas, Wen Bin Wei

**Affiliations:** ^1^Beijing Tongren Eye Center, Beijing Key Laboratory of Intraocular Tumor Diagnosis and Treatment, Beijing Tongren Hospital, Capital Medical University, Beijing, China; ^2^Beijing Ophthalmology and Visual Sciences Key Lab, Beijing Tongren Hospital, Capital Medical University, Beijing, China; ^3^Medical Artificial Intelligence Research and Verification Key Laboratory of the Ministry of Industry and Information Technology, Beijing Tongren Hospital, Capital Medical University, Beijing, China; ^4^Beijing Ophthalmology and Visual Science Key Laboratory, Beijing Tongren Eye Center, Beijing Tongren Hospital, Beijing Institute of Ophthalmology, Capital Medical University, Beijing, China; ^5^Department of Ophthalmology, Medical Faculty Mannheim, Heidelberg University, Mannheim, Germany; ^6^Institute of Molecular and Clinical Ophthalmology Basel, Basel, Switzerland

**Keywords:** neuregulin-1, epidermal growth factor, EGF, myopia, axial elongation

## Abstract

**Background:**

Since the mechanisms underlying myopic axial elongation have remained unclear, we examined the effect of neuregulin-1 (NRG-1), an epidermal growth factor family member, on myopic axial elongation.

**Methods:**

The guinea pigs aged two to three weeks were subjected to bilateral negative lens-induced axial elongation and received weekly intravitreal injections into their right eyes of NRG-1 antibody (doses: 5 μg, *n* = 8; 10 μg, *n* = 8, 20 μg, *n* = 9) or of NRG-1 (doses: 0.05 μg, *n* = 8; 0.01 μg, *n* = 9; 0.2 μg, *n* = 8), underwent only bilateral negative lens-induced axial elongation (myopia control group, *n* = 10), or underwent no intervention (control group, *n* = 10). The contralateral eyes received corresponding intravitreal phosphate-buffered solution injections. One week after the last injection, the guinea pigs were sacrificed, the eyeballs were removed, the thicknesses of the retina and sclera were histologically examined, the expression of NRG-1 and downstream signal transduction pathway members (ERK1/2 and PI3K/AKT) and the mRNA expression of NRG-1 in the retina was assessed.

**Results:**

The inter-eye difference in axial length at study end increased (*p* < 0.001) from the normal control group (−0.02 ± 0.09 mm) and the myopia control group (−0.01 ± 0.09 mm) to the low-dose NRG-1 antibody group (−0.11 ± 0.05 mm), medium-dose NRG-1 antibody group (−0.17 ± 0.07 mm), and high-dose NRG-1 antibody group (−0.28 ± 0.06 mm). The relative expression of NRG-1, ERK1/2, and PI3K/AKT in the retina decreased in a dose-dependent manner from the myopia control group to the NRG-1 antibody groups and the normal control group. The relative NRG-1 mRNA expression in the retina was higher (*p* < 0.01) in the myopic control group than in the NRG-1 antibody groups and normal control group. Scleral and retinal thickness decreased from the normal control group to the NRG-1 antibody groups to the myopic control group. After intraocular injection of NRG-1 protein, there was a slight dose-dependent increase in the difference in axial length between the right and left eye, however not statistically significantly, from the normal control group (−0.02 ± 0.09 mm) to the high-dose NRG-1 protein group (0.03 ± 0.03 mm; *p* = 0.12).

**Conclusion:**

Intravitreal NRG-1 antibody application was dose-dependently and time-dependently associated with a reduction in negative lens-induced axial elongation in young guinea pigs.

## Introduction

With the marked increases in the prevalence of axial myopia, pathologic myopia has become one of the most common causes of irreversible vision impairment and blindness worldwide (1, 2). The development and progression of myopia are characterized by axial elongation. A better understanding of the physiology and pathophysiology of axial elongation is thus warranted. In previous studies, intraocular or topical application of various molecules has been found to affect externally induced axial elongation of the eyes in various animal species, including guinea pigs. These molecules include dopamine and its agonists and antagonists (3–9), citicoline (10), fibroblast growth factor (11, 12), transforming growth factor beta (TGF-β) (13–17), hepatocyte growth factor (18), insulin-like growth factor (19), atropine (20–24), amphiregulin (25–28), and others (29, 30). Among these molecules, low-concentration atropine (0.01%) has recently been approved by the American Food and Drug Administration for the prevention of myopia progression in children and adolescents aged 3 to 17 years (20–24). Previous studies have shown that inhibiting the epidermal growth factor (EGF) receptor through the intravitreal injection of EGF receptor antibodies notably decreased the lengthening of the axis in guinea pigs (28). In recent investigations, intraocular application of antibodies against amphiregulin, EGF, and EGF receptor in young guinea pigs with lens-induced axial elongation resulted in a decrease of axial elongation, while the intravitreal application of amphiregulin or EGF itself led to an increase in axial elongation (26–28, 30). Amphiregulin belongs to the EGF family.

Neuregulin-1 (NRG-1) as another EGF family member is a ligand for multiple receptors in the EGF family and binds to erythroblastic oncogene B (ErbB) 2, ErbB3, and ErbB4. It activates the ErbB signaling pathway and its downstream signaling pathways, such as the rat sarcoma virus/extracellular signal-regulated kinase (RAS/ERK) and phosphatidylinositol-3 kinase/protein kinase B (PI3K/Akt) pathways (31, 32). Since the effect of other EGF family members, such as EGF, amphiregulin, betacellulin, epiregulin and epigen, on experimental ocular elongation has already been examined, we hypothesized that also NRG-1 may affect negative lens-induced axial elongation. We therefore conducted this mechanistic study to better understand the mechanism of myopic axial elongation, and we examined the effect of intraocularly injected NRG-1 on axial elongation in young guinea pigs.

## Methods

The experimental investigation involved 80 juvenile male guinea pigs, ranging from 2 to 3 weeks in age, and possessing a body weight within the range of 100 to 150 grams at the baseline of the study. The research conducted in this study was subject to the approval of the Ethics Committee of the Beijing Tongren Hospital, and it adhered to the principles outlined in the ARVO Statement for the Use of Animals in Ophthalmic and Vision Research. These guinea pigs were accommodated in an environment where the temperature remained constant at 26°C, and they received a regular supply of food and water. The light and dark cycles followed a 12-h pattern, with automatic transitions occurring at 8 am and 8 pm, maintaining a luminous intensity within the range of 450–500 lx.

### Groups of experimental animals

The guinea pigs were divided into 8 groups randomly:

1. The normal control group (*n* = 10) without any intervention;
2. The myopia group (*n* = 10) with binocular negative lens wear;
3. The low-dose NRG-1 antibody group (*n* = 10), in which the animals wore bilateral negative lenses and received, at weekly intervals, three intravitreal injections of NRG-1 antibody (volume 5 μL; concentration: 1 μg/μl) (Catalog Number: AF-296-NA, R&D Systems, Minneapolis, MN, USA) at a dose of 5 μg into the right eyes and corresponding intravitreal injections of phosphate-buffered solution (PBS) (volume 5 μL) into the left eyes;
4. The medium-dose NRG-1 antibody group (*n* = 10), in which the guinea pigs wore bilateral negative lenses and were given three intravitreal injections of NRG-1 antibody (10 μg, 5 μL; concentration: 2 μg/μl) into their right eyes at weekly intervals. The left eyes received intravitreal injections of PBS (volume 5 μL);
5. The high-dose NRG-1 antibody group (*n* = 10), in which the animals wore bilateral negative lenses and were given three intravitreal injections of NRG-1 antibody (20 μg, 5 μL; concentration: 4 μg/μl) into their right eyes at weekly intervals. The left eyes received intravitreal injections of PBS (volume 5 μL);
6. The low-dose NRG-1 protein group (*n* = 10), in which the animals did not wear negative lenses but received three intravitreal injections of NRG-1 protein (volume 5 μL) (Catalog Number: 9875-NR, R&D Systems, Minneapolis, MN, USA) at a dose of 0.05 μg (concentration: 0.01 μg/μl) into their right eyes at weekly intervals. The left eyes received intravitreal injections of PBS (volume 5 μL);
7. The medium-dose NRG-1 protein group (*n* = 10), in which the guinea pigs did not wear negative lenses but received three intravitreal injections of NRG-1 protein (0.1 μg, 5 μL; concentration: 0.02 μg/μl) into the right eyes at weekly intervals. The left eyes received intravitreal injections of PBS (volume 5 μL);
8. The high-dose NRG-1 protein group (*n* = 10), in which the animals did not wear negative lenses but received three intravitreal injections of NRG-1 protein (0.2 μg, 5 μL; concentration: 0.04 μg/μl) into the right eyes at weekly intervals and the left eyes received intravitreal injections of PBS (volume 5 μL).

Animals that died or that had developed an injection-related traumatic cataract or injection-related infectious endophthalmitis during the study period were excluded. The investigation ultimately included 70 guinea pigs (control group, *n* = 10; myopia group, *n* = 10; low-dose NRG-1 antibody group, *n* = 8; medium-dose NRG-1 antibody group, *n* = 8; high-dose NRG-1 antibody group, *n* = 9; low-dose NRG-1 protein group, *n* = 8; medium-dose NRG-1 protein group, *n* = 9; high-dose NRG-1 protein group, *n* = 8).

### Negative lens-induced myopization, intraocular injections, and ocular biometry

The animals underwent examinations both at the study’s baseline and at weekly intervals. For these evaluations, the animals were subjected to surface anesthesia, and ultrasound (A/B-mode scan; oscillator frequency: 11 MHz; Quantel Co., Les Ulis, France) was used to measure their ocular axial length, anterior chamber depth, lens thickness, and vitreous cavity length. The sound velocities were calibrated as 1,557.5 m/s for the cornea and aqueous humor, 1,723.3 m/s for the lens, and 1,540 m/s for the vitreous cavity (33). For subsequent statistical analysis, the mean value was calculated from a total of 10 measurements.

After these examinations, intravitreal injections were carried out in the NRG-1 antibody groups and NRG-1 protein groups. We applied antibiotic eye drops containing ofloxacin (Santen Co., Osaka, Japan) before and after each injection. Under the influence of topical anesthesia facilitated by 0.5% proparacaine hydrochloride eye drops (Alcon Co., Puurs, Belgium), intravitreal injections were administered. The injections were carried out using Hamilton microneedles (Hamilton-MicroliterTM syringe, Sigma–Aldrich, St. Louis, MO, USA). The needle was inserted 2 mm posterior to the upper limbus, with its tip oriented toward the posterior pole. The injections were administered once per week for three weeks. One day after each injection (i.e., on days 1, 8, and 15), the animals were re-examined. On day 22, which was one week following the third and final injection, the animals were euthanized.

After the baseline measurements, all guinea pigs except for those in the control group and the NRG-1 protein groups were fitted with binocular negative lenses (−10 D). The goggles were securely attached to the rims of both eyes, as explained in great detail recently (27, 28, 34). To guarantee that the guinea pigs could still open their eyes and blink without any restrictions, utmost caution was taken during the use of the goggles. The goggles underwent daily inspections to confirm their cleanliness and proper positioning. In cases where they were found to be soiled or misplaced, they were promptly removed and substituted with new goggles. For each examination and each intravitreal injection, the goggles were removed and reapplied afterward. The guinea pigs in both the normal control group and myopia group were examined and sacrificed at the corresponding time points as those for the animals in the remaining groups.

### Tissue collection

The guinea pigs were sacrificed in deep anesthesia by an intraperitoneal injection of 1,000 mg/kg urethane as described in detail previously, and the eyes were enucleated (35). For histopathological examination, the eyes of three randomly selected guinea pigs from each of the control group, the myopia group, and the NRG-1 antibody groups, were collected. The extracted eyeballs were submerged in 10 mL of FAS Eyeball Fixative Solution (Wuhan Servicebio Technology Co. Ltd., Wuhan, China) and subsequently embedded in paraffin.

For the examination of the gene expression and for Western blotting, we used the right eyes of three other guinea pigs from the control group, the myopia group, and the NRG-1 antibody groups. Under a microscope, the retina-choroid tissue was meticulously dissected after carefully removing the cornea, lens, and vitreous. Throughout this process, all procedures were conducted on ice, and the collected samples were immediately stored in liquid nitrogen prior to being transferred to a freezer set at −80°C.

### Hematoxylin–eosin (HE) and terminal deoxynucleotidyl transferase dUTP nick end labeling (TUNEL) staining

HE and TUNEL staining were performed as described previously (28). Apoptotic cells in the retinal tissue were identified through TUNEL staining using the Cell Death Detection Kit from Kaiji Biotechnology Co. Ltd., Jiangsu, China. Quantification was performed on the TUNEL-positive cells in the retina by photographing three sections from each eye. The average count from the three assessments was documented. On the histological sections, we measured the thicknesses of the retina and sclera at the posterior pole, at the equator, and close to the ora serrata under a light microscope (Olympus Co., Tokyo, Japan).

### Western blotting and real-time PCR

Western blotting and real-time PCR were performed in accordance with methods consistent with previous investigations (28). Western blotting was employed to quantify protein expression levels. Retinal-choroid tissues that were frozen were homogenized and lysed using a lysis buffer (RIPA, Amresco, Solon City, OH, USA) that was cold. The lysis buffer was supplemented with protease inhibitors (Roche, Basel, Switzerland) and phosphatase inhibitors (Roche, Basel, Switzerland). Following a conventional procedure, the tissue extracts were subsequently transferred onto nitrocellulose membranes after being separated on 8% SDS–PAGE gels. To block the membranes, a solution of 5% skim milk in TBST (Tris–HCl, NaCl, and Tween 20) was applied for a period of two hours. Subsequently, they were sequentially incubated with primary antibodies overnight and with secondary antibodies for two hours on the subsequent day. The ECL kit (Millipore, MA, USA) was used for signal detection, and TotalLab Quant V11.5 (Newcastle upon Tyne, UK) was used to capture images. Afterwards, ImageJ (NIH, Maryland, USA) was used to quantify and analyze the target bands, with glyceraldehyde-3-phosphate-dehydrogenase (GAPDH) acting as an internal control. The primary antibodies were against NRG-1 (ImmunoWay YT3054, 1:10,000), ERK1/2 (ImmunoWay YM3516, 1:20,000), phospho (p)-ERK1/2 (CST, 4377, 1:10,000), PI3K (ImmunoWay YN5491, 1:10,000), p-PI3K (ImmunoWay YP0224, 1:2,000), AKT (CST, 9272, 1:20,000), p-AKT (ImmunoWay YP0590, 1:2,000), and GAPDH (ImmunoWay YM3029, 1:20,000).

The frozen retina-choroid tissues mentioned above were utilized to analyze the relative expression of NRG-1 mRNA. TRIzol reagent (Invitrogen, CA, USA) was used to extract RNA from the retina-choroid. To determine the RNA’s purity, the UV-2450 spectrophotometer from Shimadzu, Japan was utilized to measure the optical density (OD) value. The Reverse Transcription System kit (TaKaRa, Japan) was used to perform reverse transcription on two to three micrograms of RNA. SYBR Green (TaKaRa, Japan) was used in a 20 μL reaction for quantitative real-time PCR. The reaction included 1 μL of cDNA, 10 μL of SYBR Green Mix, 7 μL of ddH2O, and 1 μL of each specific primer. The cycling parameters were set as follows: 95°C for 15 s, 60°C for 20 s, and 72°C for 40 s. The specificity of the PCR product was confirmed through melting curve analysis (28). The primers for endogenous GAPDH were as follows: forward, 5′- GGGAAGCTCACAGGTATGGC-3′, and reverse, 5′-TGTCATCGTATTTGGCCGGT-3′. The primers for endogenous NRG-1 were as follows: forward, 5′-AAAGGGCAGGAAGAAGGAGC-3′, and reverse, 5′-TGCCGCTGACTCTTGACTTT-3′. The relative expression of each gene in each intervention group as compared with the control group was determined using the 2^-ΔΔCt^ method.

We measured the relative expression of NRG-1 and the members of the downstream ERK1/2 and PI3K/AKT and NRG-1 mRNA expression in the retina to get additional information about the involvement of NRG-1 in the process of axial elongation and to explore which of the various downstream pathways after the activation of the EGF receptor by NRG-1 taken.

### Statistical analysis

Statistical analysis was performed using SPSS 27.0 software (SPSS for Windows, version 27.0, IBM-SPSS, Chicago, IL, USA) and GraphPad Prism 9.4.0 (GraphPad Software, San Diego, CA, USA). We calculated the mean ± standard deviation of the outcome parameters and assessed the normality of the distribution of the parameters using the Shapiro–Wilk test. We applied Levene’s test to assess the homogeneity of variance and paired-sample Student t tests to examine the statistical significance of the difference between the left and right eyes of the same animals. Independent-sample Student t tests were used to examine the statistical significance of differences between groups of animals. A *p* value of <0.05 was considered to indicate statistical significance.

## Results

### Effect of negative lens-induced myopia on the axial length of guinea pigs

**Figure 1:**
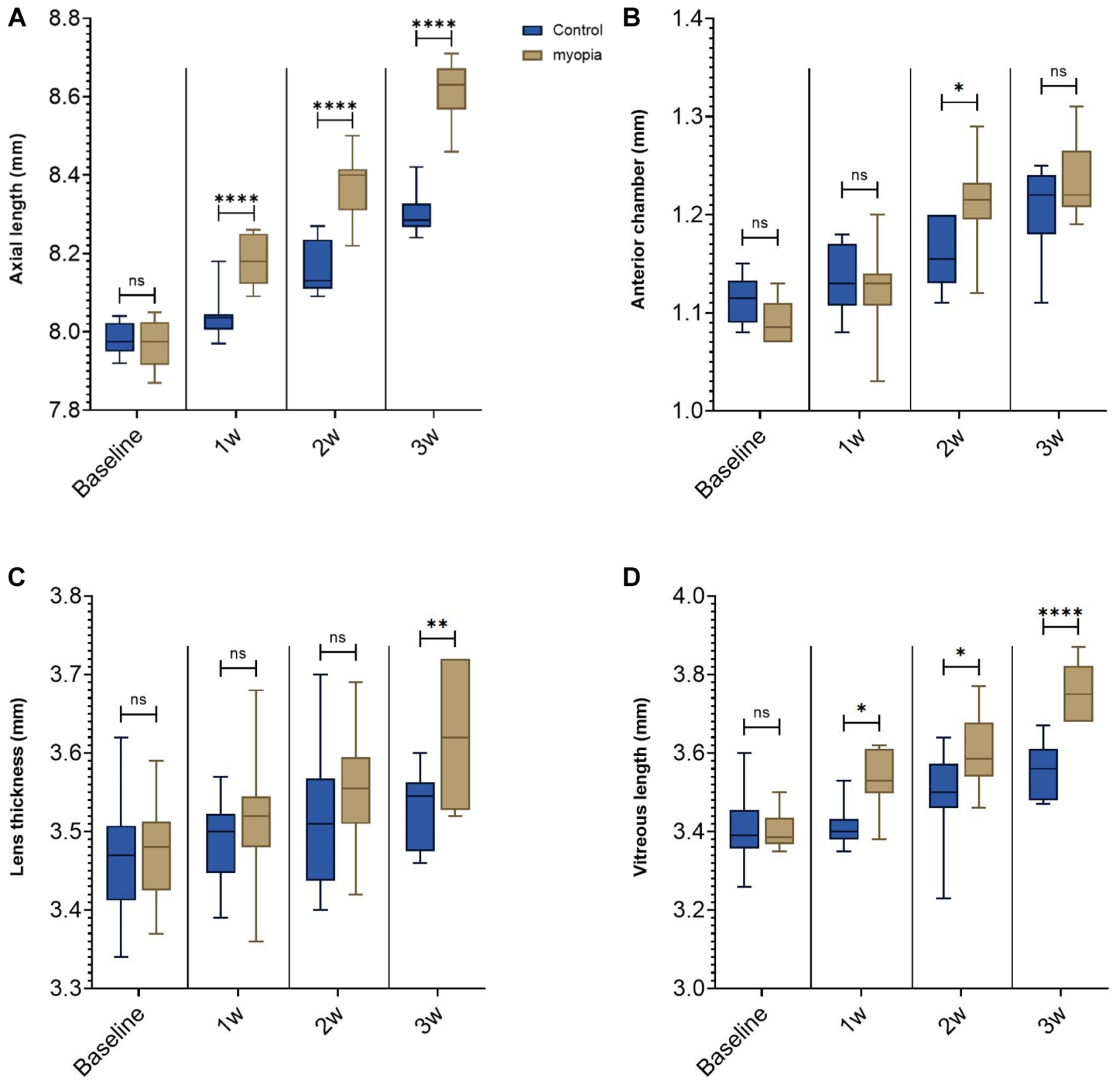
Negative lens-induced axial elongation in guinea pigs. **(A)** Axial length of the right eyes in the control group without any intervention and in the myopia group with binocular negative lens-induced myopization. **(B)** Anterior chamber depth of the right eyes in the control group without any intervention and in the myopia group with binocular negative lens-induced axial elongation at the study end. **(C)** Lens thickness of the right eyes in the control group without any intervention and in the myopia group with binocular negative lens-induced axial elongation at the study end. **(D)** Vitreous cavity length of the right eyes in the control group without any intervention and in the myopia group with binocular negative lens-induced axial elongation at the study end (*p < 0.05, **p < 0.01, ****p < 0.001).

At baseline, the biometric parameters of axial length, anterior chamber depth, lens thickness, and vitreous cavity length did not differ significantly (all *p* ≥ 0.05) between the right eyes and left eyes within each group or between the groups (Table 1). Axial elongation was significantly greater in the myopia group than in the control group (Tables 1, 2; Figure 1). The difference in axial elongation between groups increased with the length of follow-up. The difference in right-eye axial length between the myopia group and the control group was 0.14 ± 0.06 mm at one week of follow-up and increased to 0.31 ± 0.09 mm at three weeks of follow-up (Tables 1, 2). The increase in axial length was mostly due to an increase in the length of the vitreous cavity (*p* < 0.001) and was to a lesser extent due to an increase in lens thickness (*p* = 0.006); the anterior chamber depth (*p* = 0.14) did not differ significantly between the myopia group and the control group (Table 1; Figures 1B–D).

**Table 1:** Biometric measurements in each group (mean ± standard deviation).

Group	*n*	Eyes	Time	AC (mm)	LT (mm)	VL (mm)	AL (mm)
Control group	10	Right eyes	Baseline	1.11 ± 0.02	3.47 ± 0.08	3.41 ± 0.09	7.98 ± 0.04
			Study end	1.21 ± 0.04	3.53 ± 0.05	3.56 ± 0.07	8.30 ± 0.05
		Left eyes	Baseline	1.11 ± 0.03	3.5 ± 0.09	3.37 ± 0.09	7.98 ± 0.05
			Study end	1.2 ± 0.06	3.55 ± 0.08	3.58 ± 0.09	8.32 ± 0.10
Myopia group	10	Right eyes	Baseline	1.09 ± 0.02	3.48 ± 0.06	3.40 ± 0.05	7.97 ± 0.06
			Study end	1.24 ± 0.04	3.62 ± 0.08	3.76 ± 0.07	8.61 ± 0.09
		Left eyes	Baseline	1.09 ± 0.04	3.48 ± 0.07	3.41 ± 0.08	7.99 ± 0.05
			Study end	1.23 ± 0.05	3.65 ± 0.09	3.75 ± 0.09	8.62 ± 0.08
Low-dose NRG-1 antibody group	8	Right eyes	Baseline	1.09 ± 0.03	3.47 ± 0.05	3.45 ± 0.07	8.02 ± 0.04
			Study end	1.23 ± 0.01	3.61 ± 0.1	3.68 ± 0.08	8.51 ± 0.07
		Left eyes	Baseline	1.06 ± 0.04	3.47 ± 0.09	3.47 ± 0.08	8.00 ± 0.06
			Study end	1.22 ± 0.01	3.63 ± 0.1	3.75 ± 0.08	8.61 ± 0.09
Medium dose NRG-1 antibody group	8	Right eyes	Baseline	1.08 ± 0.04	3.48 ± 0.09	3.44 ± 0.09	7.99 ± 0.06
			Study end	1.25 ± 0.03	3.62 ± 0.07	3.57 ± 0.08	8.43 ± 0.10
		Left eyes	Baseline	1.07 ± 0.05	3.51 ± 0.06	3.43 ± 0.06	8.00 ± 0.05
			Study end	1.22 ± 0.03	3.65 ± 0.09	3.73 ± 0.09	8.60 ± 0.06
High-dose NRG-1 antibody group	9	Right eyes	Baseline	1.07 ± 0.04	3.53 ± 0.08	3.39 ± 0.07	7.99 ± 0.06
			Study end	1.22 ± 0.01	3.62 ± 0.04	3.52 ± 0.05	8.36 ± 0.07
		Left eyes	Baseline	1.07 ± 0.05	3.5 ± 0.07	3.43 ± 0.04	8.00 ± 0.09
			Study end	1.23 ± 0.04	3.65 ± 0.1	3.77 ± 0.08	8.64 ± 0.08
Low-dose NRG-1 protein group	8	Right eyes	Baseline	1.08 ± 0.04	3.52 ± 0.06	3.36 ± 0.07	7.96 ± 0.06
			Study end	1.22 ± 0.02	3.58 ± 0.06	3.59 ± 0.11	8.39 ± 0.06
		Left eyes	Baseline	1.1 ± 0.03	3.48 ± 0.11	3.36 ± 0.09	7.97 ± 0.05
			Study end	1.21 ± 0.03	3.59 ± 0.08	3.59 ± 0.08	8.38 ± 0.05
Medium dose NRG-1 protein group	9	Right eyes	Baseline	1.11 ± 0.02	3.49 ± 0.06	3.39 ± 0.07	7.99 ± 0.05
			Study end	1.19 ± 0.01	3.56 ± 0.07	3.62 ± 0.1	8.36 ± 0.06
		Left eyes	Baseline	1.11 ± 0.03	3.48 ± 0.1	3.38 ± 0.09	7.97 ± 0.04
			Study end	1.2 ± 0.02	3.55 ± 0.09	3.61 ± 0.11	8.35 ± 0.04
High-dose NRG-1 protein group	8	Right eyes	Baseline	1.11 ± 0.02	3.49 ± 0.05	3.39 ± 0.08	7.98 ± 0.06
			Study end	1.21 ± 0.03	3.57 ± 0.09	3.60 ± 0.04	8.37 ± 0.06
		Left eyes	Baseline	1.11 ± 0.03	3.47 ± 0.1	3.39 ± 0.09	7.97 ± 0.06
			Study end	1.22 ± 0.03	3.53 ± 0.1	3.60 ± 0.08	8.34 ± 0.06

AC, anterior chamber; LT, lens thickness; VL, vitreous length; AL, axial length.

**Table 2:** Mean increase in axial length during the study period in each group (mean ± standard deviation) (mm).

Group	Right eyes	Left eyes	*P* value
Control group (No myopization, no injections)	0.32 ± 0.07	0.34 ± 0.13	0.59
Myopia group (Myopization, no injections)	0.64 ± 0.10	0.64 ± 0.09	0.91
Low-dose NRG-1 antibody group (Myopization, injections of NRG-1 antibody (5 μg))	0.49 ± 0.08	0.62 ± 0.10	0.017
Medium dose NRG-1 antibody group (Myopization, injections of NRG-1 antibody (10 μg))	0.44 ± 0.14	0.59 ± 0.10	0.025
High-dose NRG-1 antibody group (Myopization, injections of NRG-1 antibody (20 μg))	0.37 ± 0.07	0.65 ± 0.13	<0.001
Low-dose NRG-1 protein group (Injections of NRG-1 (0.05 μg), no myopization)	0.43 ± 0.05	0.42 ± 0.08	0.79
Medium dose NRG-1 protein group (Injections of NRG-1 (0.01 μg), no myopization)	0.38 ± 0.07	0.38 ± 0.05	0.91
High-dose NRG-1 protein group (Injections of NRG-1 (0.2 μg), no myopization)	0.39 ± 0.10	0.37 ± 0.10	0.78

### Inhibition of negative lens-induced axial elongation with an NRG-1 antibody

**Figure 2:**
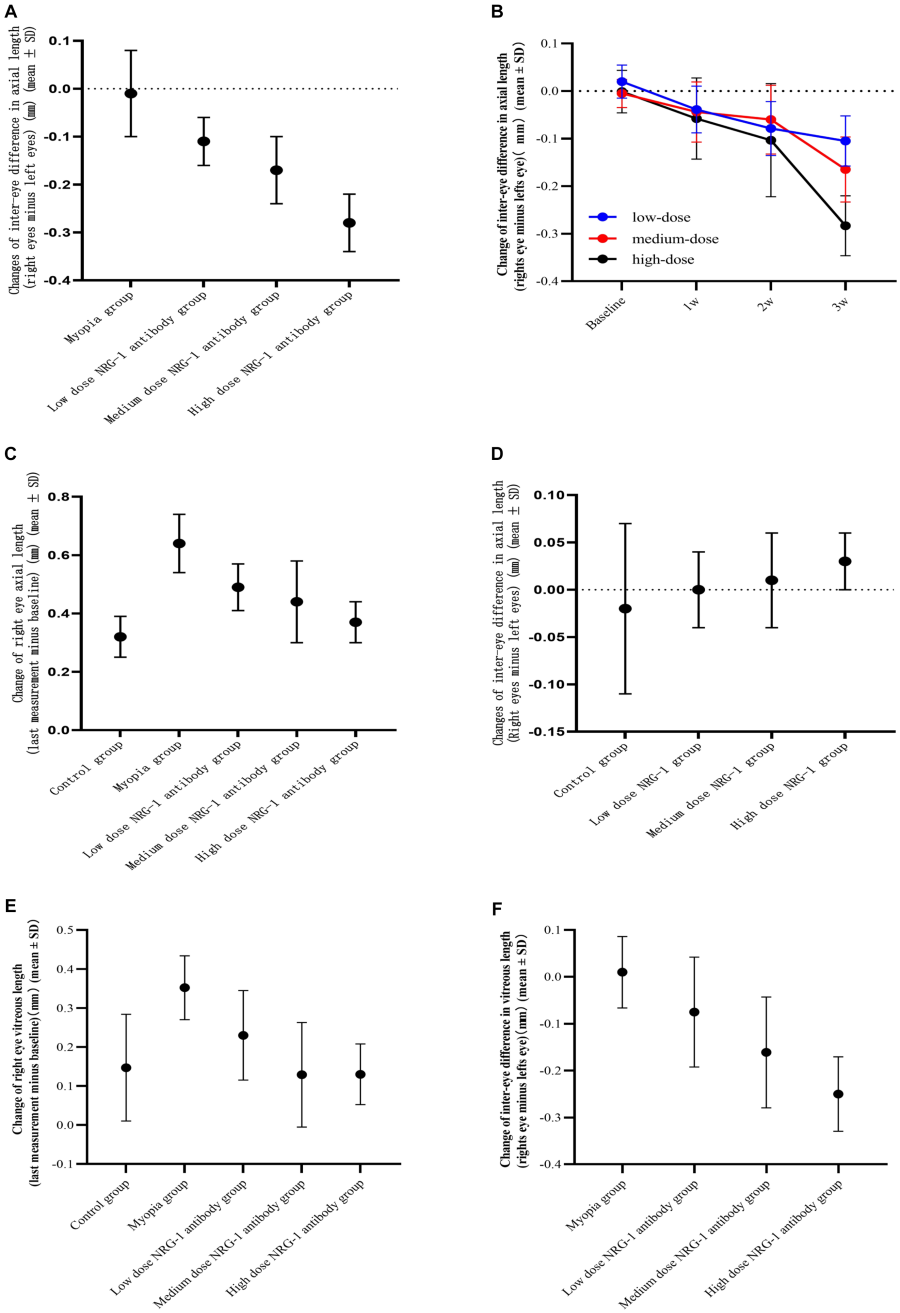
Inter-eye difference (right eye minus left eye) in axial length. **(A)** Inter-eye difference in axial length at study end in guinea pigs of the myopia group (binocular lens-induced myopization) and guinea pigs of the groups with intravitreal injections of NRG-1 antibody at various doses. **(B)** Inter-eye difference in axial length in guinea pigs of the various dose NRG-1 antibody groups during the study period. **(C)** Axial elongation in the right eyes at study end of guinea pigs of the myopia group (binocular lens-induced myopization) and guinea pigs of the groups with intravitreal injections of NRG-1 antibody at various doses. **(D)** Inter-eye difference in axial length at study end in guinea pigs of the control group and guinea pigs of the groups with intravitreal injections of NRG-1 protein at various doses. **(E)** Elongation of the vitreous cavity in the right eyes of guinea pigs at the end of the follow-up. **(F)** Inter-eye difference in vitreous length in guinea pigs at the end of follow-up.

At the end of the treatment period, the right-eye axial length in the NRG-1 antibody group was significantly lower than that in the myopia group (myopia group: 8.61 ± 0.09 mm; low-dose NRG-1 antibody group: 8.51 ± 0.07 mm, *p* = 0.01; medium-dose NRG-1 antibody group: 8.43 ± 0.10 mm, *p* = 0.001; high-dose NRG-1 antibody group: 8.36 ± 0.07 mm, *p* < 0.001) (Table 1). The difference in axial length between the right eyes and left eyes increased with higher doses of injected NRG-1 antibody and with a higher number of intraocular injections (Table 1; Figures 2A,B). After three intravitreal injections, the axial elongation in the right eyes compared with the left eyes was significantly reduced in the NRG-1 antibody groups (low-dose NRG-1 antibody group: *p* = 0.017; medium-dose NRG-1 group: *p* = 0.025; high-dose NRG-1 antibody group: *p* < 0.001). Correspondingly, axial elongation in the right eyes during the study period was greatest in the myopia group (0.64 ± 0.10 mm), followed by the low-dose NRG-1 antibody group (0.49 ± 0.08 mm) and the medium-dose NRG-1 antibody group (0.44 ± 0.14 mm); it was lowest in the high-dose NRG-1 antibody group (0.37 ± 0.07 mm) (Table 2; Figure 2C). The inter-eye difference in axial length at the study end increased (*p* < 0.001) from the normal group (−0.02 ± 0.09 mm) and the myopia control group (−0.01 ± 0.09 mm) to the low-dose NRG-1 antibody group [−0.11 ± 0.05 mm; *p* = 0.014 (in comparison to the myopia group)], medium-dose NRG-1 antibody group [−0.17 ± 0.07 mm; *p* = 0.001 (in comparison to the myopia group)], and high-dose NRG-1 antibody group [−0.28 ± 0.06 mm; *p* < 0.001 (in comparison to the myopia group)]. There was no significant difference (all *p* > 0.05) in the axial length of the left eye among the groups (Table 1).

The differences in axial length between the groups were due to changes in the vitreous cavity length (Table 1). The increase in the vitreous cavity length (the last measurement minus the baseline measurement) during the study period was significantly greater in the myopia group than in the control group (*p* = 0.001). The increases in vitreous cavity length were significantly smaller in the NRG-1 antibody groups than in the myopia group, and these attenuation effects were dose-dependent (Table 1; Figure 2E). The differences in vitreous cavity length between the right eyes and left eyes were significantly and dose-dependently elevated in the NRG-1 antibody groups compared with the myopia group (low-dose NRG-1 antibody group: *p* = 0.08; medium-dose NRG-1 antibody group: *p* = 0.002; high-dose NRG-1 antibody group: *p* = 0 < 0.001) (Table 1; Figure 2F). The differences in the anterior chamber depth and lens thickness between the right eyes and left eyes did not differ significantly between the NRG-1 antibody groups and the myopia group (all *p* > 0.05).

For the NRG-1 protein groups, the inter-eye difference in axial length (right eye minus left eye) increased, although not significantly, from the normal control group (inter-eye difference: −0.02 ± 0.09 mm) to the low-dose NRG-1 protein group (0.00 ± 0.04 mm; *p* = 0.49), medium-dose NRG-1 protein group (0.01 ± 0.05 mm; *p* = 0.35), and high-dose NRG-1 protein group (0.03 ± 0.03 mm; *p* = 0.12) (comparisons conducted with the normal control group) (Table 1; Figure 2D). The differences in axial length between the right eyes and the left eyes did not differ significantly within the NRG-1 protein groups (Tables 1, 2).

### Retinal and scleral thickness and NRG-1 antibodies

**Figure 3:**
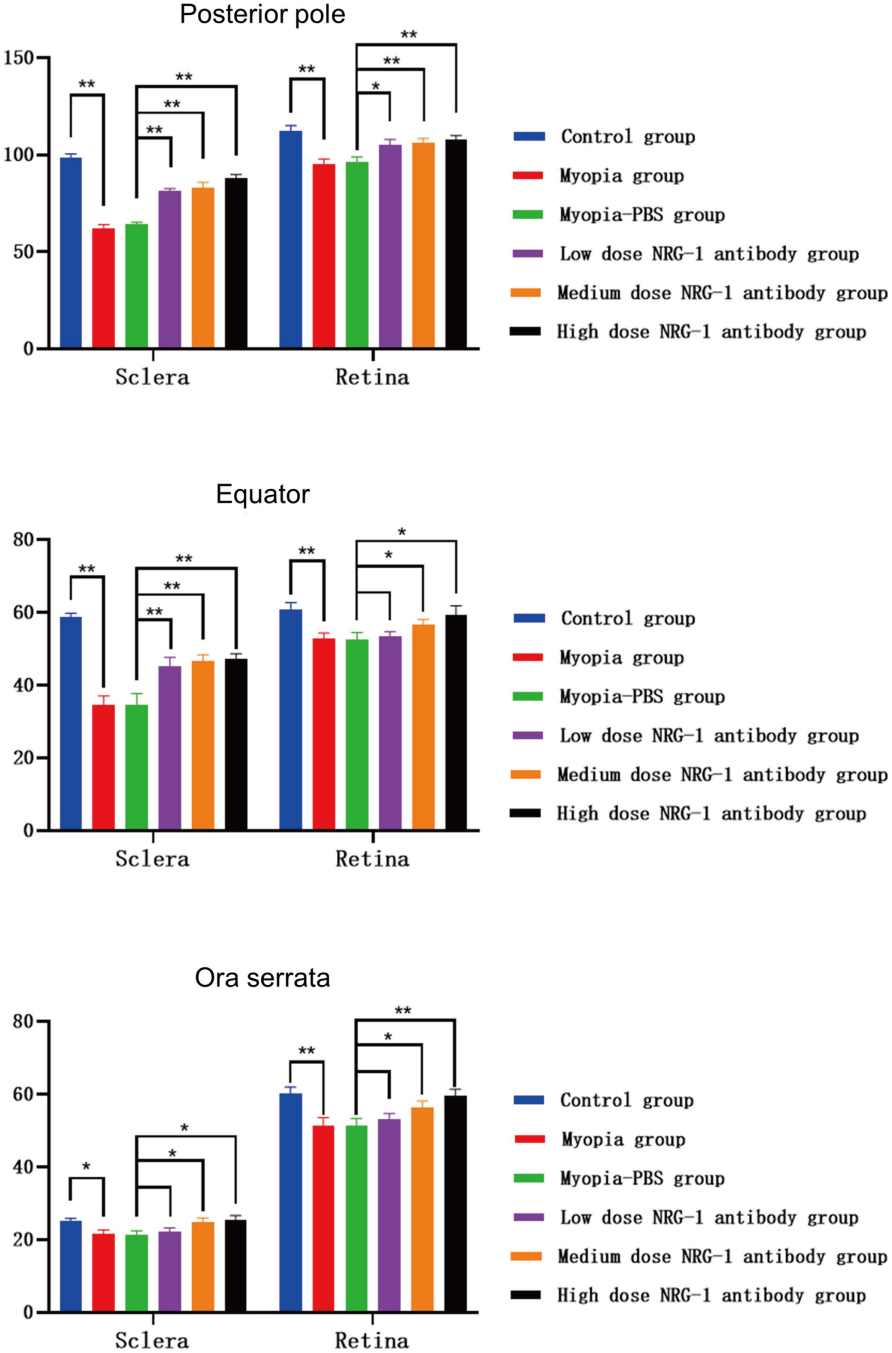
Thicknesses of the sclera and retina at the posterior pole, at the equator, and near the ora serrata in guinea pigs in each group (μm) (*p < 0.05, **p < 0.01).

Histomorphometry revealed that the scleral thickness in the control group was greatest at the posterior pole (98.4 ± 2.2 μm), followed by the equator (58.6 ± 1.2 μm), while it was the lowest at the ora serrata (25.1 ± 0.8 μm). The retina was the thickest at the posterior pole (112.0 ± 3.0 μm) and did not differ significantly between the equator (60.9 ± 1.8 μm) and the ora serrata (60.1 ± 1.9 μm). The thickness of the sclera and retina at the posterior pole, at the equator, and close to the ora serrata were significantly lower in the myopia group than in the control group (all *p* < 0.05) (Figure 3). In both, the myopia group and the various doses of NRG-1 antibody groups, the scleral thickness exhibited the same pattern, with the thickest sclera at the posterior pole, followed by the equator, and the thinnest at the ora serrata, showing significant differences in scleral thickness among these different locations. As for the retina, in all groups, the posterior pole exhibited the thickest retina, while there were no significant differences in retinal thickness between the equator and the ora serrata (Figure 3).

Comparing the NRG-1 antibody groups with the myopia group, both the sclera and retina were thicker at the posterior pole (*p* ≤ 0.001 and *p* = 0.10, respectively), and the sclera was also significantly thicker at the equator (*p* = 0.006), in the low-dose NRG-1 antibody group than in the myopia group. When comparing the medium and high-dose NRG-1 antibody groups with the myopia group, the retina and sclera, measured at the posterior pole, equator and ora serrata, were significantly thicker in the medium and high-dose NRG-1 antibody groups than in the myopia group (Figure 3). Scleral and retinal thickness increased significantly in a dose dependent manner from the myopia group to the NRG-1 antibody groups (Figure 3).

**Figure 4:**
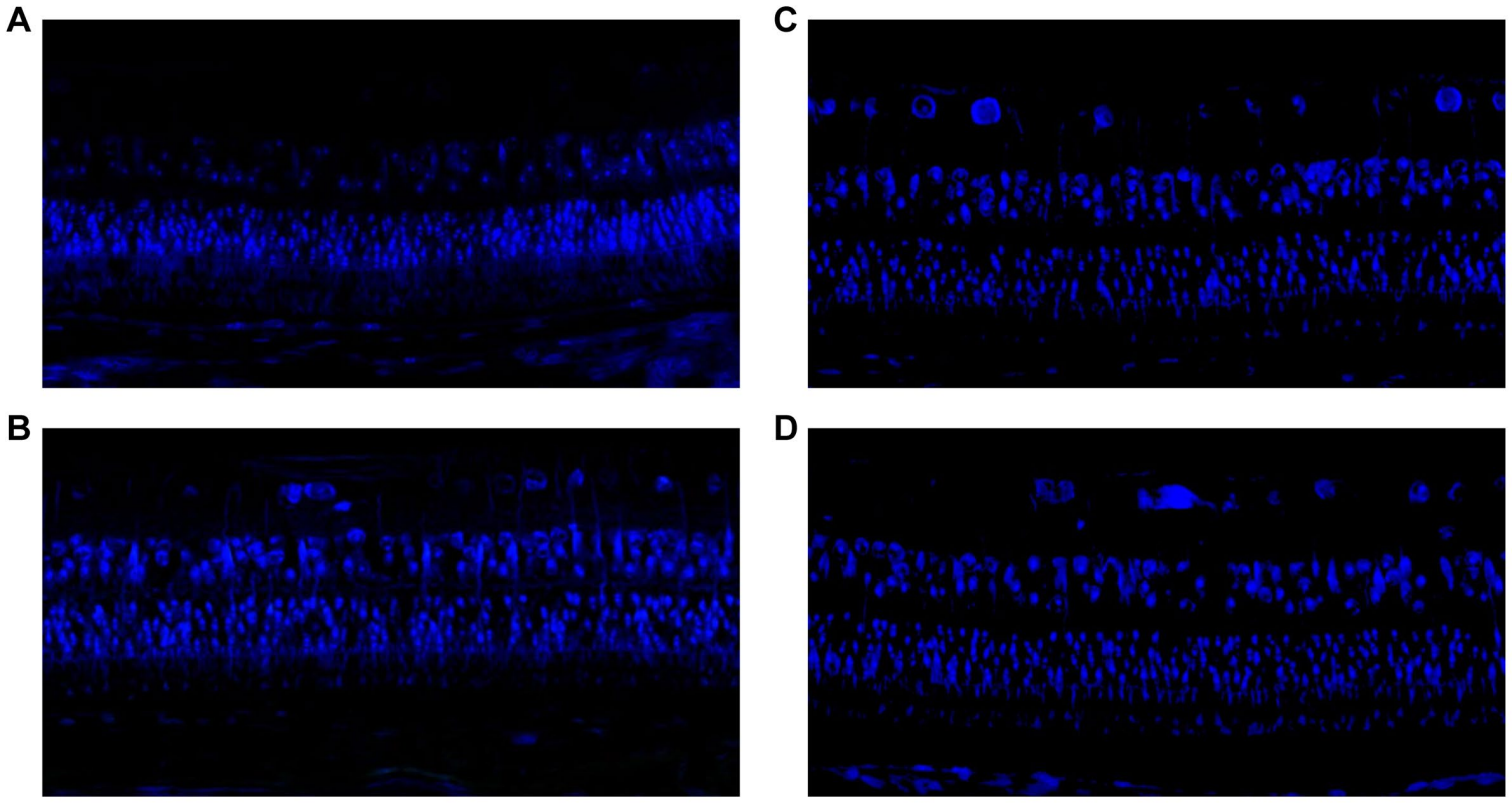
**(A)** TUNEL staining in guinea pigs with lens-induced myopia but without any intravitreal applications. **(B)** TUNEL staining in guinea pigs with lens-induced myopia combined with intravitreal applications of 20 μg of NRG-1 antibody. **(C)** TUNEL staining in guinea pigs without lens-induced myopia or any intravitreal applications. **(D)** TUNEL staining in guinea pigs with intravitreal applications of 0.2 μg of NRG-1 protein but without lens-induced myopia.

Examination of the TUNEL-stained slides revealed no significant differences in the counts of TUNEL-positive cells at the inner nuclear layer and outer nuclear layers between the eyes that received intravitreal NRG-1 antibody injections and the myopia-only eyes. Similarly, there was no significant differences in the counts of TUNEL-positive cells between the normal control group and the NRG-1 protein groups (Figure 4).

### Inhibition of protein and gene expression in the NRG-1 antibody groups

**Figure 5:**
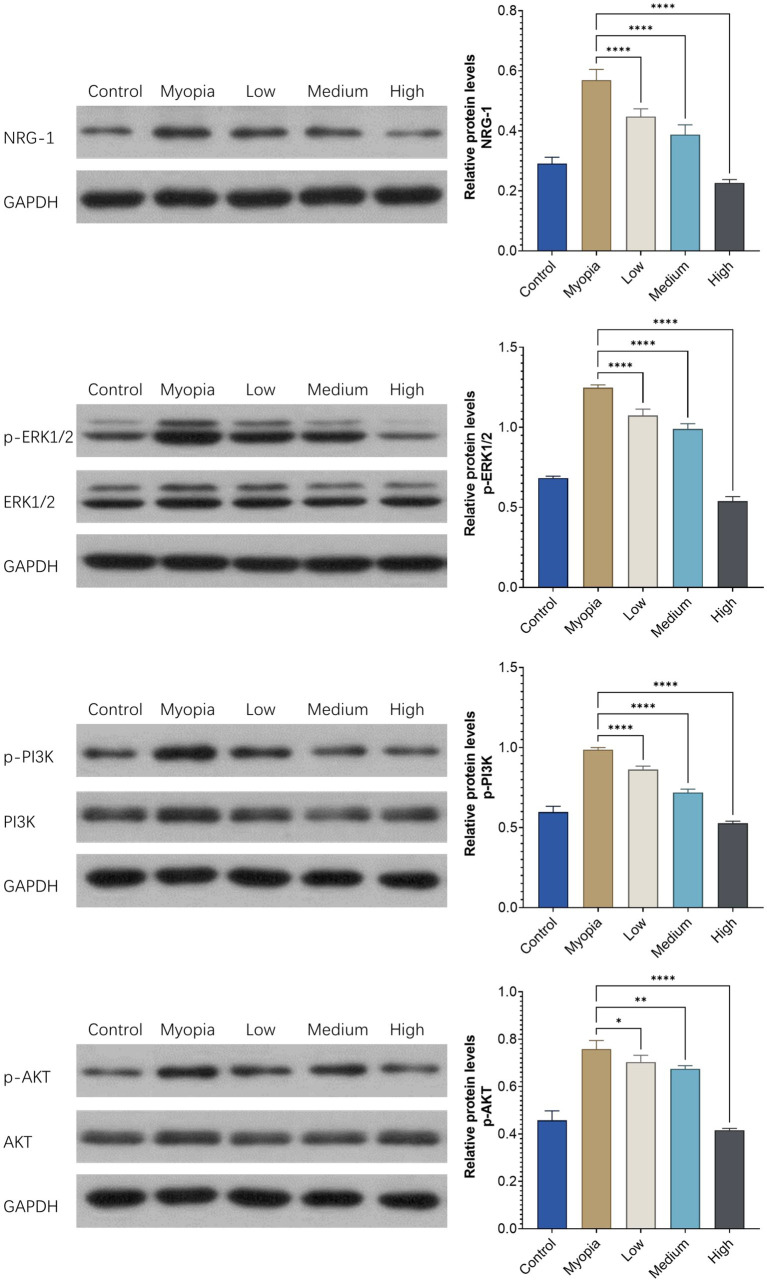
The relative expression levels of NRG-1 and downstream proteins in the retinas of young guinea pigs were assessed across different experimental conditions: those without any intervention (“Control”), those with bilateral lens-induced myopia (“Myopia”), and those with bilateral lens-induced myopia receiving repeated intraocular applications of NRG-1 antibody at doses of 5 μg (“Low”), 10 μg (“Medium”), and 20 μg (“High”) into the right eye, while intravitreal applications of PBS were administered into the left eye. The provided *p* values indicate the statistical significance of differences when compared to the group of guinea pigs with bilateral lens-induced myopia (“Myopia”) (*p < 0.05, **p < 0.01, ****p < 0.001).

The relative expression of NRG-1 and the members of the downstream ERK1/2 and PI3K/AKT signal transduction pathways in the retina-choroid tissue was examined by Western blotting. The relative expression levels of p-ERK1/2, p-PI3K, and p-AKT were higher (all *p* < 0.05) in the myopia group than in the control group. In the NRG-1 antibody groups, the relative expression of p-ERK1/2, p-PI3K, and p-AKT was reduced in a dose-dependent manner (Figure 5).

**Figure 6:**
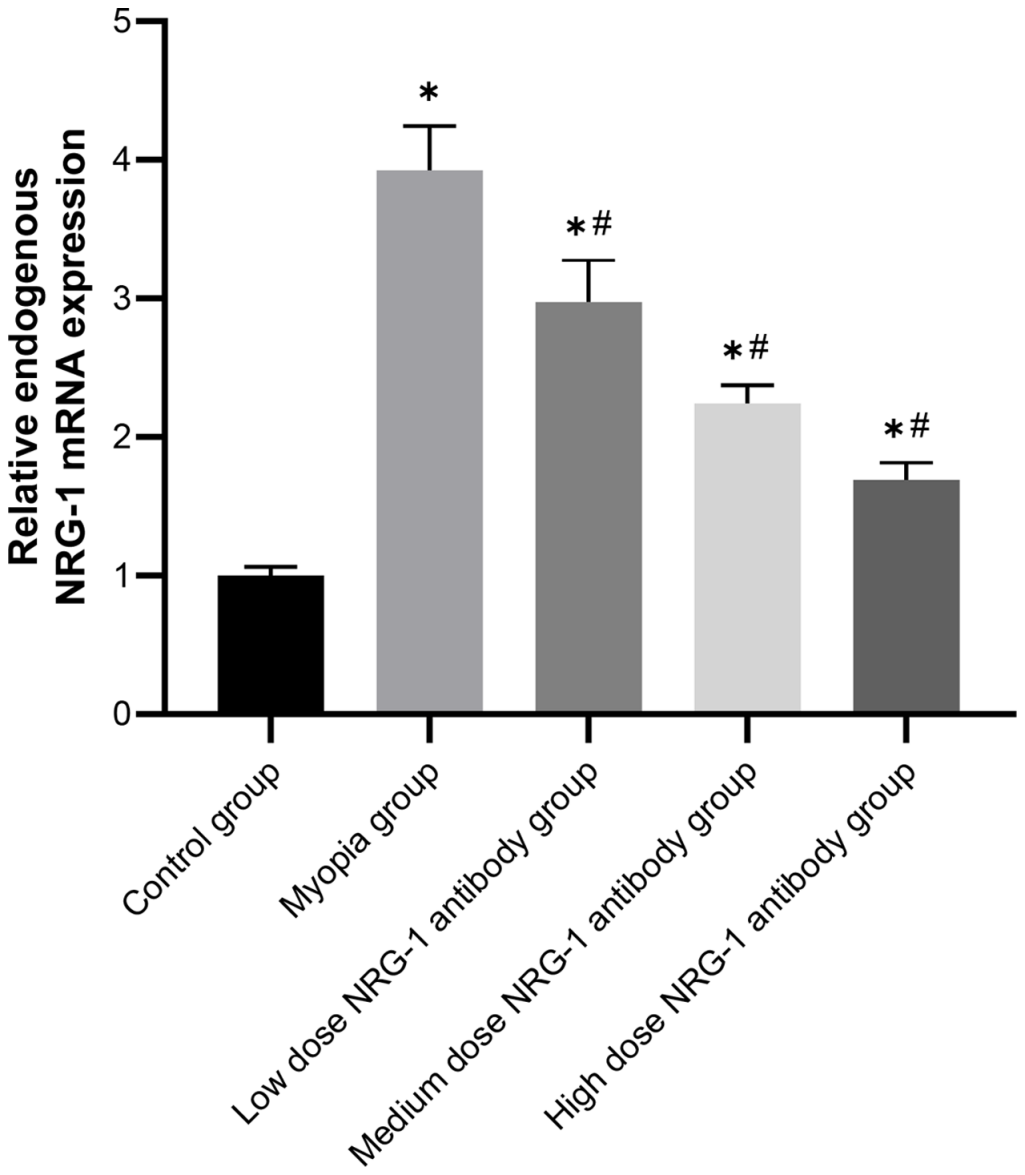
Relative expression of NRG-1 mRNA in the retinas of guinea pigs in different groups. *p < 0.05 compared to the control group, #*p* < 0.05 compared to the myopia group.

Real-time PCR was used to examine NRG-1 mRNA expression in the retina. The mRNA expression of NRG-1 in control retinas was standardized to 1 to calculate the relative expression of NRG-1 mRNA in the retinas of the other groups. The relative retinal NRG-1 mRNA expression was significantly higher in the myopia group than in the control group (*p* < 0.01). In the NRG-1 antibody groups, the relative expression of NRG-1 mRNA was significantly lower than that in the myopia group (*p* < 0.01). The expression of NRG-1 mRNA gradually decreased with increasing doses of intraocularly injected NRG-1 antibody (Figure 6).

## Discussion

The longitudinal intra-eye comparison and inter-eye comparison of the current study revealed that bilateral negative lens-induced axial elongation in young guinea pigs was significantly lower in the eyes intravitreally injected with NRG-1 antibody than in the contralateral eyes intravitreally injected with PBS injections. In addition, the axial elongation was lower in the eyes of young guinea pigs intravitreally injected with NRG-1 antibody than in the eyes of young guinea pigs with negative lens-induced axial elongation but without intraocular injections. These effects were dose-dependent and time-dependent. The differences in axial length between the NRG-1 antibody groups and the myopia group were due to changes in the vitreous cavity length, while the between-side differences in the anterior chamber depth and the lens thickness did not differ significantly (all *p* > 0.05) between the NRG-1 antibody groups and the myopia group. In guinea pigs with unilateral intravitreal injections of NRG-1 protein, the differences between the right eyes and the left eyes increased slightly, but not statistically significantly (*p* = 0.49, *p* = 0.35, *p* = 0.12, respectively) (Figure 2D). The differences in axial length between the groups were paralleled by differences in scleral and retinal thickness (Figure 3). Activation of downstream signaling pathways of ErbB, such as ERK1/2 and PI3K/AKT, in the retina-choroid tissue of guinea pigs was linked to the occurrence of negative lens-induced axial elongation. In contrast, intraocular injections of NRG-1 antibody were correlated with significant and dose-dependent reductions in ERK1/2 and PI3K/AKT activation as well as attenuated axial elongation. The relative NRG-1 mRNA expression in the retina was higher in the myopia group than in the control group. After intravitreal injection of NRG-1 antibody, the relative expression of NRG-1 mRNA was reduced in a dose-dependent manner.

The observations made in our study agree with and extend findings obtained in previous investigations. Research conducted by Jiang and his team, as well as Dong and his colleagues, has shown that intravitreal injections of antibodies against amphiregulin (another member of the EGF family), EGF, and EGF receptor are associated with dose-related and time-dependent decreases in negative lens-induced axial elongation in young guinea pigs and that intravitreally applied amphiregulin or EGF itself leads to an increase in axial elongation (26–28). Our study extends these observations to NRG-1, another member of the EGF family.

NRG-1 is a cell adhesion molecule encoded in humans by the NRG1 gene (36, 37). NRG-1 is one of four proteins in the neuregulin family that acts on the EGF family of receptors. It is produced in numerous isoforms by alternative splicing. The various functions of NRG-1 include influences on the normal development of the nervous system and the heart (38). Inside the eye, NRG-1 is expressed mainly in the retinal ganglion cell layer and inner nuclear layer and can promote nerve regeneration (39, 40). Associations of NRG-1 with ocular axial elongation and axial myopia have not yet been described.

In our study, the reduction in axial elongation associated with the intraocularly applied NRG-1 antibody was due primarily to lengthening of the vitreous cavity (Figure 1). This agrees with the findings in other studies in which axial elongation affected mostly the length of the vitreous cavity (3–16). The changes in the thickness of the sclera and retina in eyes with a change in axial length observed in this study are in agreement with the outcomes of prior clinical and experimental research (26–28).

Previous studies have shown that NRG-1 binds to multiple receptors of the EGF family and activates the ErbB signaling pathway and its downstream PI3K/AKT, MAPK, and other signaling pathways (31, 32, 41). Correspondingly, in our study, the relative expression of the downstream signaling pathway members p-ERK1/2, p-PI3K, and p-AKT was higher in the myopia group than in the control group, while it was decreased in a dose-dependent manner in the NRG-1 antibody groups (Figure 5). These findings further suggest that NRG-1 was associated with axial elongation in our study and that the NRG-1 induced activation of the EGF receptor led to an activation of both primary downstream signaling pathways of the EGF receptor, i.e., the PI3K/Akt/PTEN/mTOR pathway and the RAS/RAF/MEK/ERK pathway.

After the intraocular injection of the NRG-1 protein, axial elongation increased in a dose dependent manner slightly, but not statistically significantly, from the normal control group to the high-dose NRG-1 protein group. The reason for the stronger blocking effect on axial elongation by the NRG-1 antibody as compared to the axial elongation increasing effect by the NRG-1 protein have remained unclear. The relatively small number of animals included into the study may have reduced the statistical power to show a statistically significant effect for the NRG-1 protein. In contrast, the intravitreal application of amphiregulin, another EGF family member, has been found to lead to a significant increase in axial elongation in young guinea pigs in a previous study (27).

Our study has several limitations that warrant discussion. Firstly, while our experimental study has established an association between NRG-1 signaling and axial elongation, the specifics of the downstream pathway remain unclear. Given that the complex regulatory network of myopic axial elongation includes the retina, choroid and sclera, future investigations are warranted to examine the downstream pathway of NRG-1. Second, we did not perform refractometry and keratometry, so our investigation was based on alterations and differences in axial length. Comparing the biometric readings to refractometric measurements could have yielded more pertinent findings for our investigation goal, as the axial length and axial elongation biometric parameters are the most significant factors associated with complications related to myopia. Thirdly, it’s important to note that the sample sizes in our study were relatively small, which may have limited the statistical power. However, this limitation could also potentially contribute to reinforcing the conclusion that intravitreally administered NRG-1 antibodies effectively mitigate axial elongation in young guinea pigs with bilateral negative lens-induced axial elongation. Fourth, it’s worth acknowledging that histomorphometric measurements of retinal and scleral thickness could have been affected by various factors, such as alterations in blood vessel saturation prior to and after enucleation, swelling of tissues following enucleation, shrinkage of tissues due to fixation, and the presence of other artifacts related to the preparation of histological slides. Fifth, it’s important to consider that the type of goggles used for myopic axial elongation might have had an impact on the process of axial elongation. The −10-diopter goggles employed in our study could yield different effects compared to −4.0-diopter goggles or diffuser lenses, potentially introducing variability in the results.

In conclusion, intraocular application of neuregulin-1 antibodies was associated with dose-dependent reductions in axial elongation in the eyes of young guinea pigs with negative lens-induced myopization. These reductions were paralleled by protein expression, gene expression, and morphometric changes. In contrast, intraocular injection of NRG-1 protein increased axial elongation.

## Data availability statement

The raw data supporting the conclusions of this article will be made available by the authors, without undue reservation.

## Ethics statement

The Ethics Committee of the Beijing Tongren Hospital study approved the study. The study was conducted in accordance with the local legislation and institutional requirements.

## Author contributions

XS: Conceptualization, Data curation, Formal analysis, Investigation, Methodology, Validation, Visualization, Writing – original draft. LD: Conceptualization, Investigation, Methodology, Validation, Writing – original draft. RZ: Investigation, Validation, Writing – review & editing. WZ: Investigation, Validation, Writing – review & editing. YFL: Investigation, Writing – review & editing. HW: Investigation, Writing – review & editing. HL: Investigation, Writing – review & editing. CY: Investigation, Writing – review & editing. YTL: Investigation, Writing – review & editing. YW: Conceptualization, Methodology, Writing – review & editing. JJ: Conceptualization, Methodology, Writing – review & editing. WW: Conceptualization, Funding acquisition, Writing – review & editing, Methodology.

## Abbreviations

NRG-1: Neuregulin-1
TGF-β: Transforming growth factor beta
EGF: Epidermal growth factor
ErbB: Erythroblastic oncogene B
RAS: Rat sarcoma virus
ERK: Extracellular signal-regulated kinase
PI3K: Phosphatidylinositol-3 kinase
Akt: Protein kinase B
PBS: Phosphate-buffered solution
HE: Hematoxylin–eosin
TUNEL: Terminal deoxynucleotidyl transferase dUTP nick end labeling

## References

[1] Holden BA Fricke TR Wilson DA Jong M Naidoo KS Sankaridurg P. Global prevalence of myopia and high myopia and temporal trends from 2000 through 2050. Ophthalmology. (2016) 123:1036–42. doi: 10.1016/j.ophtha.2016.01.006, PMID: 26875007

[2] Xu L Wang Y Li Y Wang Y Cui T Li J. Causes of blindness and visual impairment in urban and rural areas in Beijing: the Beijing eye study. Ophthalmology. (2006) 113:1134.e1–1134.e11. doi: 10.1016/j.ophtha.2006.01.035 PMID: 16647133

[3] Mao J Liu S Qin W Li F Wu X Tan Q. Levodopa inhibits the development of form-deprivation myopia in Guinea pigs. Optom Vis Sci. (2010) 87:53–60. doi: 10.1097/OPX.0b013e3181c12b3d 19901858

[4] Gao Q Liu Q Ma P Zhong X Wu J Ge J. Effects of direct intravitreal dopamine injections on the development of lid-suture induced myopia in rabbits. Graefes Arch Clin Exp Ophthalmol. (2006) 244:1329–35. doi: 10.1007/s00417-006-0254-1, PMID: 16550409

[5] Yan T Xiong W Huang F Zheng F Ying H Chen J-F. Daily injection but not continuous infusion of Apomorphine inhibits form-deprivation myopia in mice. Invest Ophthalmol Vis Sci. (2015) 56:2475–85. doi: 10.1167/iovs.13-12361, PMID: 25744977

[6] McCarthy CS Megaw P Devadas M Morgan IG. Dopaminergic agents affect the ability of brief periods of Normal vision to prevent form-deprivation myopia. Exp Eye Res. (2007) 84:100–7. doi: 10.1016/j.exer.2006.09.018, PMID: 17094962

[7] Lan W Yang Z Feldkaemper M Schaeffel F. Changes in dopamine and Zenk during suppression of myopia in chicks by intense illuminance. Exp Eye Res. (2016) 145:118–24. doi: 10.1016/j.exer.2015.11.018 26657138

[8] Jiang L Long K Schaeffel F Zhou X Zheng Y Ying H. Effects of dopaminergic agents on progression of naturally occurring myopia in albino Guinea pigs (*Cavia Porcellus*). Invest Ophthalmol Vis Sci. (2014) 55:7508–19. doi: 10.1167/iovs.14-14294, PMID: 25270191

[9] Mao J Liu S Fu C. Citicoline retards myopia progression following form deprivation in Guinea pigs. Exp Biol Med (Maywood). (2016) 241:1258–63. doi: 10.1177/1535370216638773 26979720 PMC4950317

[10] Wu X-H Li Y-Y Zhang P-P Qian K-W Ding J-H Hu G. Unaltered retinal dopamine levels in a C57bl/6 mouse model of form-deprivation myopia. Invest Ophthalmol Vis Sci. (2015) 56:967–77. doi: 10.1167/iovs.13-13362, PMID: 25604682

[11] Rohrer B Stell WK. Basic fibroblast growth factor (BFGF) and transforming growth factor Beta (TGF-Beta) act as stop and go signals to modulate postnatal ocular growth in the Chick. Exp Eye Res. (1994) 58:553–61. doi: 10.1006/exer.1994.1049, PMID: 7925692

[12] Seko Y Shimokawa H Tokoro T. Expression of bFGF and TGF-Beta 2 in experimental myopia in chicks. Invest Ophthalmol Vis Sci. (1995) 36:1183–7. PMID: 7730028

[13] Jobling AI Wan R Gentle A Bui BV McBrien NA. Retinal and choroidal TGF-Beta in the tree shrew model of myopia: isoform expression, activation and effects on function. Exp Eye Res. (2009) 88:458–66. doi: 10.1016/j.exer.2008.10.022 19046968

[14] McBrien NA. Regulation of scleral metabolism in myopia and the role of transforming growth factor-Beta. Exp Eye Res. (2013) 114:128–40. doi: 10.1016/j.exer.2013.01.014 23399866

[15] Li X-J Yang X-P Wan G-M Wang Y-Y Zhang J-S. Effects of hepatocyte growth factor on MMP-2 expression in scleral fibroblasts from a Guinea pig myopia model. Int J Ophthalmol. (2014) 7:239–44. doi: 10.3980/j.issn.2222-3959.2014.02.09 24790864 PMC4003076

[16] Tian X-D Cheng Y-X Liu G-B Guo S-F Fan C-L Zhan L-H. Expressions of type I collagen, Α2 integrin and Β1 integrin in sclera of Guinea pig with defocus myopia and inhibitory effects of bFGF on the formation of myopia. Int J Ophthalmol. (2013) 6:54–8. doi: 10.3980/j.issn.2222-3959.2013.01.11, PMID: 23550266 PMC3580250

[17] Cheng T Wang J Xiong S Zhang B Li Q Xu X. Association of IGF1 single-nucleotide polymorphisms with myopia in Chinese children. PeerJ. (2020) 8:e8436. doi: 10.7717/peerj.8436, PMID: 32025377 PMC6991122

[18] Jia Y Hu D-N Zhou J. Human aqueous humor levels of TGF- Β2: relationship with axial length. Biomed Res Int. (2014) 2014:258591. doi: 10.1155/2014/258591 24967344 PMC4055366

[19] Chen B-Y Wang C-Y Chen W-Y Ma J-X. Altered TGF-Β2 and BFGF expression in scleral Desmocytes from an experimentally-induced myopia Guinea pig model. Graefes Arch Clin Exp Ophthalmol. (2013) 251:1133–44. doi: 10.1007/s00417-013-2269-8 23381656

[20] Flitcroft DI. The complex interactions of retinal, optical and environmental factors in myopia aetiology. Prog Retin Eye Res. (2012) 31:622–60. doi: 10.1016/j.preteyeres.2012.06.004 22772022

[21] Yen MY Liu JH Kao SC Shiao CH. Comparison of the effect of atropine and Cyclopentolate on myopia. Ann Ophthalmol. (1989) 21:180–7. PMID: 2742290

[22] Chua W-H Balakrishnan V Chan Y-H Tong L Ling Y Quah B-L. Atropine for the treatment of childhood myopia. Ophthalmology. (2006) 113:2285–91. doi: 10.1016/j.ophtha.2006.05.062 16996612

[23] Chia A Lu Q-S Tan D. Five-year clinical trial on atropine for the treatment of myopia 2: myopia control with atropine 0.01% Eyedrops. Ophthalmology. (2016) 123:391–9. doi: 10.1016/j.ophtha.2015.07.004 26271839

[24] Yam JC Li FF Zhang X Tang SM Yip BHK Kam KW. Two-year clinical trial of the low-concentration atropine for myopia progression (lamp) study: phase 2 report. Ophthalmology. (2020) 127:910–9. doi: 10.1016/j.ophtha.2019.12.011, PMID: 32019700

[25] Huang J Wen D Wang Q McAlinden C Flitcroft I Chen H. Efficacy comparison of 16 interventions for myopia control in children: a network meta-analysis. Ophthalmology. (2016) 123:697–708. doi: 10.1016/j.ophtha.2015.11.010, PMID: 26826749

[26] Jiang WJ Song HX Li SY Guo B Wu JF Li GP. Amphiregulin antibody and reduction of axial elongation in experimental myopia. EBioMedicine. (2017) 17:134–44. doi: 10.1016/j.ebiom.2017.02.021, PMID: 28256400 PMC5360597

[27] Dong L Shi XH Kang YK Wei WB Wang YX Xu XL. Amphiregulin and ocular axial length. Acta Ophthalmol. (2019) 97:e460–70. doi: 10.1111/aos.14080, PMID: 30860674

[28] Dong L Shi XH Li YF Jiang X Wang YX Lan YJ. Blockade of epidermal growth factor and its receptor and axial elongation in experimental myopia. FASEB J. (2020) 34:13654–70. doi: 10.1096/fj.202001095RR, PMID: 32799354

[29] Barathi VA Weon SR Beuerman RW. Expression of muscarinic receptors in human and mouse sclera and their role in the regulation of scleral fibroblasts proliferation. Mol Vis. (2009) 15:1277–93. PMID: 19578554 PMC2704914

[30] Wang W Nan Y Huang T Pu M Jonas JB. Intraocular Amphiregulin antibody and axial elongation in nonhuman Primates. Front Ophthalmol. (2022):2. doi: 10.3389/fopht.2022.995157 PMC11182130 38983534

[31] Chen X Shen J Zhou Q Jin X Liu H Gao R. Astragaloside vi ameliorates post-stroke depression via upregulating the NRG-1-mediated MEK/ERK pathway. Pharmaceuticals (Basel). (2022) 15:1551. doi: 10.3390/ph15121551 36559001 PMC9784132

[32] Fang S-J Wu X-S Han Z-H Zhang X-X Wang C-M Li X-Y. Neuregulin-1 preconditioning protects the heart against ischemia/reperfusion injury through a PI3k/Akt-dependent mechanism. Chin Med J. (2010) 123:3597–604. doi: 10.1007/s00380-008-1094-1, PMID: 22166638

[33] Zhou X Qu J Xie R Wang R Jiang L Zhao H. Normal development of refractive state and ocular dimensions in Guinea pigs. Vis Res. (2006) 46:2815–23. doi: 10.1016/j.visres.2006.01.027, PMID: 16723148

[34] Dong L Shi XH Kang YK Wei WB Wang YX Xu XL. Bruch's membrane thickness and retinal pigment epithelium cell density in experimental axial elongation. Sci Rep. (2019) 9:6621. doi: 10.1038/s41598-019-43212-8, PMID: 31036950 PMC6488581

[35] Dong L Zhang R-H Wu H-T Li H-Y Zhou W-D Shi X-H. Intravitreal short-hairpin RNA attenuated adeno-associated virus-induced knockdown of Amphiregulin and axial elongation in experimental myopia. Invest Ophthalmol Vis Sci. (2023) 64:11. doi: 10.1167/iovs.64.4.11, PMID: 37040096 PMC10103729

[36] Holmes WE Sliwkowski MX Akita RW Henzel WJ Lee J Park JW. Identification of Heregulin, a specific activator of P185erbb2. Science. (1992) 256:1205–10. doi: 10.1126/science.256.5060.1205, PMID: 1350381

[37] Orr-Urtreger A Trakhtenbrot L Ben-Levy R Wen D Rechavi G Lonai P. Neural expression and chromosomal mapping of Neu differentiation factor to 8p12-P21. Proc Natl Acad Sci U S A. (1993) 90:1867–71. doi: 10.1073/pnas.90.5.1867, PMID: 8095334 PMC45981

[38] Britsch S. The Neuregulin-I/ERBB signaling system in development and disease. Adv Anat Embryol Cell Biol. (2007) 190:1–65. PMID: 17432114

[39] Yang W Liu T-T Song X-B Zhang Y Li Z-H Hao Q. Neuregulin-1 protects against acute optic nerve injury in rat model. J Neurol Sci. (2015) 357:157–66. doi: 10.1016/j.jns.2015.07.023, PMID: 26235969

[40] Klaassen I de Vries EW Vogels IMC van Kampen AHC Bosscha MI Steel DHW. Identification of proteins associated with clinical and pathological features of proliferative diabetic retinopathy in vitreous and Fibrovascular membranes. PLoS One. (2017) 12:e0187304. doi: 10.1371/journal.pone.0187304, PMID: 29095861 PMC5667868

[41] Rosas D Raez LE Russo A Rolfo C. Neuregulin 1 gene (NRG1). A potentially new targetable alteration for the treatment of Lung Cancer. Cancers (Basel). (2021) 13:5038. doi: 10.3390/cancers13205038 34680187 PMC8534274

